# Supplementary material for: Heterogeneity of multiple sclerosis lesions in fast diffusional kurtosis imaging
Source: PLoS One. 2021 Feb 4;16(2):e0245844. doi: 10.1371/journal.pone.0245844 (PMC7861404; doi:10.1371/journal.pone.0245844)
Supplement: Attachment — Submitted filename: PLOS_one_11.21.20_fyu.docx [file pone.0245844.s002.docx]

Herein, the authors acquired a fast DKI sequence in patients with relapsing remitting multiple sclerosis, and found that lesions with different imaging characteristics on conventional sequences (FLAIR, T1w, contrast-enhanced T1w) demonstrated significant differences in mean kurtosis, which was not detected utilizing their DKI-derived mean diffusivity metric. The conclusions of the paper are that the fast DKI sequence could potentially be leveraged as a tool to further replace gadolinium-enhanced imaging. The merits of this paper are its easy to acquire sequence that is superior to conventional DWI/DTI-based methods, as well as the overall thorough methods used for image analysis. Although it is clear that further work (additional subjects) are needed to validate this strategy, it is nevertheless a worthwhile effort.

1. Rather unbalanced patient and control population – would be worth commenting on; did you test for normal distribution (i.e. Kolmogorov-Smirnov test)? Also, please comment on the uneven sex distribution between the MS and control groups.
2. In addition to dimethyl fumarate, were any MS patients on other MS medication (DMARDS)? Please comment.
3. With regards to your acquisition, did you consider utilizing your newer 1-9-9 compensated acquisition? It would be worth discussing why you opted for the 1-3-9 acquisition instead, as both are efficient acquisitions?
4. Did you obtain a reversed phase-encoding direction b0 image for topup correction? If not, please indicate this limitation.
5. In the methods section, what is “rice-floor adjustment” in your postprocessing step?
6. In the methods section 2.4, you are referring to four different areas of white matter damage (as many would also consider NAWM as you’ve listed).
7. When creating the NAWM mask, in subtracting out the CE-L, did you base it on the neuroradiologists’ segmented contrast enhancing masks, or the LST segmentations (which would be more encompassing of the lesion dimensions on FLAIR)? Please comment.
8. Is there a reason you did not specifically include the temporal lobe in your NAWM ROIs?
9. Is there a reason you did not calculate the lesion fractional anisotropy and instead opted to compare to MD? If this data is available, it would be reasonable to present it.
10. In the discussion section, I think you may be misrepresenting the performance of QSM “However, in a meta-analysis by Gupta et al. [7], only FA was a robust parameter to differentiate between enhancing and non-enhancing lesions in MS patients, implicating a need for additional imaging markers to evaluate white matter and lesional damage.“ The article itself stated that QSM had the best performance for discriminating between enhancing and non-enhancing lesions, albeit with limited data.
11. In the discussion section, as to why MK did not show difference between HC and NAWM, it could also be the result of specificity of the metric to pathologic changes within the NAWM (it does not discriminate between myelin loss and axonal loss) as well as potential uncharacterized differences between your subject groups compared to other studies. I would suggest bringing up these points in your discussion.
12. An important note is that enhancing lesions are not necessarily acute, as some older lesions can enhance as well (recurrent active demyelination). Please indicate if this was something you took into consideration in your analysis (reviewed multiple comparison studies to determine the actual approximate age of the lesions, particularly the enhancing ones).
13. It would be worthwhile to comment on alternative diffusion imaging schemes, such as NODDI, and SMT. See reference: Yu, F. *et al.* Imaging G-Ratio in Multiple Sclerosis Using High-Gradient Diffusion MRI and Macromolecular Tissue Volume. *AJNR Am J Neuroradiol* **40**, 1871-1877, doi:10.3174/ajnr.A6283 (2019).
